# Joint rushing alters internal timekeeping in non-musicians and musicians

**DOI:** 10.1038/s41598-022-05298-5

**Published:** 2022-01-24

**Authors:** Thomas Wolf, Günther Knoblich

**Affiliations:** Department of Cognitive Science, Central European University, 1100 Vienna, Austria

**Keywords:** Psychology, Human behaviour

## Abstract

Recent studies have shown that people engaging in joint rhythmic activity unintentionally increase their tempo. The same tempo increase does not occur when the same rhythmic activity is performed alone. This phenomenon is known as joint rushing. In two experiments, we investigated whether joint rushing is caused by correction mechanisms that facilitate sensorimotor synchronization. Because such correction mechanisms require perceptual input, joint rushing should discontinue when auditory feedback in a joint rhythmic activity is interrupted. This prediction was clearly supported in two experiments, one with musicians and one with non-musicians. Surprisingly, there was no indication that the amount of joint rushing differed between musicians and non-musicians. Furthermore, neither musicians nor non-musicians were able to return to the initially instructed tempo after feedback had been interrupted. This result indicates that joint rushing has a lasting effect on an internal timekeeper. An important question for future research is whether joint rushing is only a dysfunctional side effect of the way sensorimotor synchronization works or whether it has a function in enabling precise temporal coordination between different individuals.

## Introduction

Humans have an astounding propensity to engage in joint action. Many joint actions, such as shaking hands, carrying objects together, engaging in a conversation, or dancing together require that the partners participating in the joint action achieve precise temporal coordination of their individual contributions to a joint action^1–4^. For discrete movements, an important timing parameter that needs to be aligned is the speed at which individual actions are performed to achieve synchronous outcomes. Previous research indicates that increasing the speed of actions can help with achieving temporal coordination, presumably because increased speed reduces the variability of actions, thereby making them more predictable for a partner^5^.

For rhythmic joint actions, such as walking together or making music together, individuals can align the tempo of their successive actions to achieve coordination^6^. Adhering to the same tempo can facilitate coordination because tempo can establish a common timing reference for partners performing rhythmic joint actions. However, this will only work if all partners involved are able to keep a certain tempo. Although tempo changes were often treated as a “methodological inconvenience”^7^ in research on temporal coordination, recent research has started to address the question of whether joint action partners can successfully keep a particular tempo or whether joint action leads to systematic tempo changes. Recent studies^8–10^ have provided converging evidence that performing rhythmic joint actions leads to a systematic tempo increase—joint rushing—which is absent in individual performance. Different proposals have been made to explain how joint rushing emerges from the interaction of individual timing systems reciprocally adapting to one another. The present study aims to provide further empirical evidence that speaks to assumptions that have been made about the contributions of continuous perceptual input, internal time keeping, and musical expertise to the emergence of joint rushing. We start with a short overview of mechanisms that could contribute to explaining joint rushing.

### Error correction mechanisms in sensorimotor synchronization

Previous studies on time keeping in individuals have addressed the ability to synchronize actions with periodic cues. It has been found that similar error correction mechanisms are used, irrespective of whether the periodic cue comes from a perfectly isochronous metronome or from another human with variable timing^11^. The two most prominent error correction mechanisms are phase correction and period correction (for a review see^12^). Phase correction is largely automatic and compensates for errors of relative phase, i.e., the timing offset between two actions but leaves the general tempo unchanged. There are two different ways in which the error signal for phase correction is formalized. In some models^13,14^ the error signal is based on asynchronies between an external timer (metronome) and a person’s actions. Here phase correction adjusts the timing based on the previous asynchrony to reduce the asynchrony between the next action and the next timing signal. In other models^15–17^ the error signal is computed as the difference in phase. The onset of a target signal corresponds to phase zero and can be compared to a person’s current phase. The difference is used to immediately adjust that person’s current phase. In this case phase correction can already lead to the adjustment of the timing of an action in the current interval.

The second mechanism of error correction is period correction. This is an intentional adjustment of the tempo specified in an internal timekeeper to align it with the tempo of a perceived periodic target sequence. As with phase correction the error signal of period correction can be calculated in two different ways. In some models^13,15^ the error signal is based on the previous asynchrony as described above for phase correction. In other models^14^ the error signal comes from a comparison between the length of the previous interval in a target sequence and the timing interval at which the internal timekeeper is currently operating. When period correction is based on the asynchrony between action and timing signal then adjustments are made for the subsequent interval^13^. However, when period correction is based on a comparison between target intervals and internally set intervals adjustments can already occur in the current interval^16,17^.

### Models of joint rushing

In conventional models of sensorimotor synchronization^13,14,18,19^ both phase and period correction produce symmetrical adjustments for positive and negative asynchronies. Hence, whether an action was too early or too late determines the direction of adjustment, but not its magnitude. However, recent studies on joint rushing have provided converging evidence that when humans synchronize to one another rather than to an external timekeeper, there is a systematic increase in tempo. At first glance, this seems to pose a problem for models that use phase and period correction to explain joint rushing^10,16,17^. How can these models account for the directionality of joint rushing with mechanisms that predict symmetrical adjustments for positive and negative asymmetries?

Okano et al.^17^ and Konvalinka et al.^16^ propose a model in which the error assessment of phase and period correction is not dependent on asynchronies in previous intervals, but rather on the phase and angular velocity in the current interval. As pointed out above, this allows for immediate adjustments in the current interval. Applied to interpersonal coordination this leads to an asymmetry in adjustments for the two interaction partners producing timing signals for each other. According to this model, if two participants tap together, they adjust their timing immediately when the other’s tap is perceived. The late tapper perceives the early tapper’s tap as timing signal and immediately adjusts by increasing phase and angular velocity, which leads the early tapper to adjust for less when she finally perceives the late tapper’s tap. Hence in each period the late tapper, who corrects first, speeds up more than the early tapper, who corrects later, slows down. Together this results in an overall speed-up of the joint performance.

Wolf et al.^10^ proposed a model of joint rushing that combines phase and period correction mechanisms based on asynchronies. To account for the directionality of joint rushing this model introduces a correction mechanism, which is inherently biased towards a shortening of periods. This phase advancement mechanism has previously been used by evolutionary biologists to explain large scale displays of synchronization observed in chorusing katydids or synchronizing fireflies^20–24^. Phase advancement is a local error correction that works by shortening concurrent periods when a partner’s signal is received in a certain time window before one’s own action is carried out. Combining phase advancement with period correction also predicts the overall speed-up in joint performance observed during joint rushing.

### The current study

The current study aims to empirically test two key assumptions that are shared by current models of joint rushing that have not been directly addressed in previous research. The first assumption is that joint rushing depends on perceptual input from the partner. All error correction mechanisms discussed above rely on continuous input from a target signal produced by a partner. Hence, if joint rushing emerges from correction mechanisms that rely on continuous perceptual input, we should see that joint rushing stops when the perceptual input stops. Alternatively joint rushing could be caused by psychophysical states such as arousal or anxiety^17,25^ that depend on the actual or imagined presence of another person rather than specific perceptual input.

The second assumption we put to test is that joint rushing involves a lasting period correction affecting an internal timekeeper. Both Okano et al.^17^ and Konvalinka et al.^16^ assume that timing adjustments during joint rhythmic performance involve period correction. In the Wolf et al.^10^ model period correction is crucial to transform local phase advancements into a lasting tempo change. However, there are also reasons to assume that period correction may not be involved in joint rushing. First, experiments by Repp and Keller^26^ show that period correction is an intentional process that requires attention and is triggered when tempo mismatches are perceptible. In contrast to these earlier findings, joint rushing often occurs at a rate that is below the level of perceptible tempo changes^27^ and it does not seem to exhibit the stepwise adjustments that are characteristic for period correction^28^. Second, most joint rushing data exhibits a characteristic flattening in tempo increase over time^8–10^. Thomson et al.^9^ model their rushing data with a piecewise regression of which the second piece is modelled as a perfectly horizontal line that implies the assumption of no rushing. A possible explanation for this plateau could be that internal timekeeping remains at the initial target tempo, which would imply no period correction. Once the difference between the current execution tempo and the internally represented target tempo becomes large enough to be detectable a further tempo increase can be prevented.

To address these questions, we invited pairs of participants to perform a dyadic, synchronization-continuation, tapping task which has already been successfully used to study joint rushing^8,10^. In the synchronization phase at the beginning of each trial the task was to synchronize with a metronome specifying the target tempo. In an ensuing continuation phase during which the metronome remained silent participants were asked to continue tapping at the rate of the specified target tempo. For the Solo condition, where the task was performed alone, we expected no increase in tempo because participants did not perceive external timing signals that supposedly cause joint rushing. For the Joint condition where participants were instructed to keep the prespecified tempo we expected to see the characteristic tempo increase observed for joint rushing.

The crucial manipulation in the current study was that in half of the trials the bidirectional auditory feedback in the joint condition stopped halfway through a trial—Joint Interrupted condition. If joint rushing depends on continuous perceptual input from a partner there should be no further tempo increase once the perceptual input stops. If joint rushing depends on the psychophysical states of the performers^25^ or if partners use internal models to simulate the timing of their partner’s taps based on the previously perceived taps^29–31^, then we should see that joint rushing continues, at least to some degree, after perceptual input stops.

If joint rushing depends on perceptual input from the partner, we can address the second question of whether joint rushing involves a change of period in an internal timekeeper. If joint rushing affects the period of an internal timekeeper, the increased tempo should be retained after perceptual input from the partner stops. If, joint rushing does not affect the period of an internal timekeeper, we expect participants to return to the initial tempo after perceptual input from the partner stops.

To address the role of expertise in joint rushing, we conducted the same experiment twice, once with a sample of non-musicians (Experiment 1) and once with a sample of musicians (Experiment 2). Comparing joint rushing across different levels of musical expertise can provide clues as to whether and how experts can avoid joint rushing. Previous evidence on this issue is contradictory. While conductors lament the tendency of orchestras to rush^32^, Thomson et al.^9^ found that joint rushing was absent in a group of musicians and suggest that musicians’ lower timing errors and timing variability may allow them to avoid joint rushing. For the current study, this would predict that musicians exhibit less joint rushing than non-musicians. A further prediction can be derived from the observation that musicians have superior abilities of sensorimotor synchronization and auditory imagery^33^. For the present task, this implies that musicians may be better able to keep imagining the target tempo during a period of joint rushing. Accordingly, musicians should be better able than non-musicians to return to the originally instructed tempo after perceptual input from their partner stops.

## Methods—Experiment 1

### Participants

We invited 24 non-musicians (13 women, 11 men, mean age = 27 years, SD = 6 years, mean years of musical training = 0 years, SD = 0 years) to participate in Experiment 1. The sample size was determined with the help of a power analysis based on pilot data from 6 participants with a desired power of 0.9 at a significance threshold of p = 0.05. This sample size was also specified during pre-registration (https://aspredicted.org/blind.php?x=s74pk3). Participants gave their informed consent and received a monetary compensation. All experiments in this study were conducted in accordance with the Declaration of Helsinki and approved by the Psychological Research Ethics Board (PREBO) at CEU, Vienna, Austria.

### Apparatus

Each participant tapped on a Sensel Morph device placed in a separate room and received auditory feedback through headphones. The experiment was controlled with a custom Max MSP patch running on a MacBook Pro, which handled among other things the production of metronome sounds, switching between the auditory feedback conditions, and recording participants’ taps. For one participant tapping on their device produced a piano sound at 155.6 Hz (D#3). For the other participant tapping produced a piano sound at 370.0 Hz (F#4).

### Procedure and design

Participants were tested in pairs. After a short introduction and general explanation participants were led to two separate rooms. The experiment was controlled by an experimenter in a third room. Each trial started with 10 s of metronome clicks at a tempo of 120 bpm, which is equivalent to an inter-click-interval of 500 ms. Participants were instructed to listen to the first four clicks and then to start tapping along with the metronome. After 10 s the metronome faded away and participants were instructed to maintain the target tempo of the metronome until they heard a stop signal after 60 s. These durations were carefully selected based on previous experiments (Wolf et al., 2019) to provide optimal sensitivity for testing our predictions. Participants were familiarized with this procedure in two shorter practice trials. In the Joint and the Joint Interrupted condition participants were instructed to not only maintain the target tempo but to also synchronize with their partner as long as the partner produced sounds.

The test phase consisted of six blocks of six trials each. The first and last block consisted of Solo trials, in which participants could only hear their own sounds throughout the whole trial. Blocks 2–4 consisted of Joint or Joint Interrupted trials. In which participants could hear their own as well as their partners’ sounds. In Joint trials this was the case for the full 60 s of the trial. In Joint Interrupted trials participants could hear the sounds produced by their partner for 30 s. The bidirectionality of the auditory feedback was stopped so that participants only heard themselves. Each joint block consisted of three uninterrupted and three interrupted trials in semi-random order following a Latin-square design. In total, each participant performed 12 Solo trials, 12 Joint trials and 12 Joint Interrupted trials.

### Analysis

The target inter-tap-interval (ITI) was 500 ms. In order to remove missing taps and double taps, we excluded all ITIs outside a 500 +—50% range from further analysis. This amounted to a removal of 1.58% of all ITIs in Experiment 1 and 1.77% of all ITIs in Experiment 2. Analyses and plots were computed on Tempo Change, which was calculated by subtracting from all ITIs the mean ITI in the synchronization phase (Time Bin 0). Negative numbers for the Tempo Change therefore indicate that the tempo in a certain Time Bin was faster than the initial tempo. All ANOVA results for factors with more than two levels were Greenhouse–Geisser corrected.

## Results—Experiment 1

**Figure 1:**
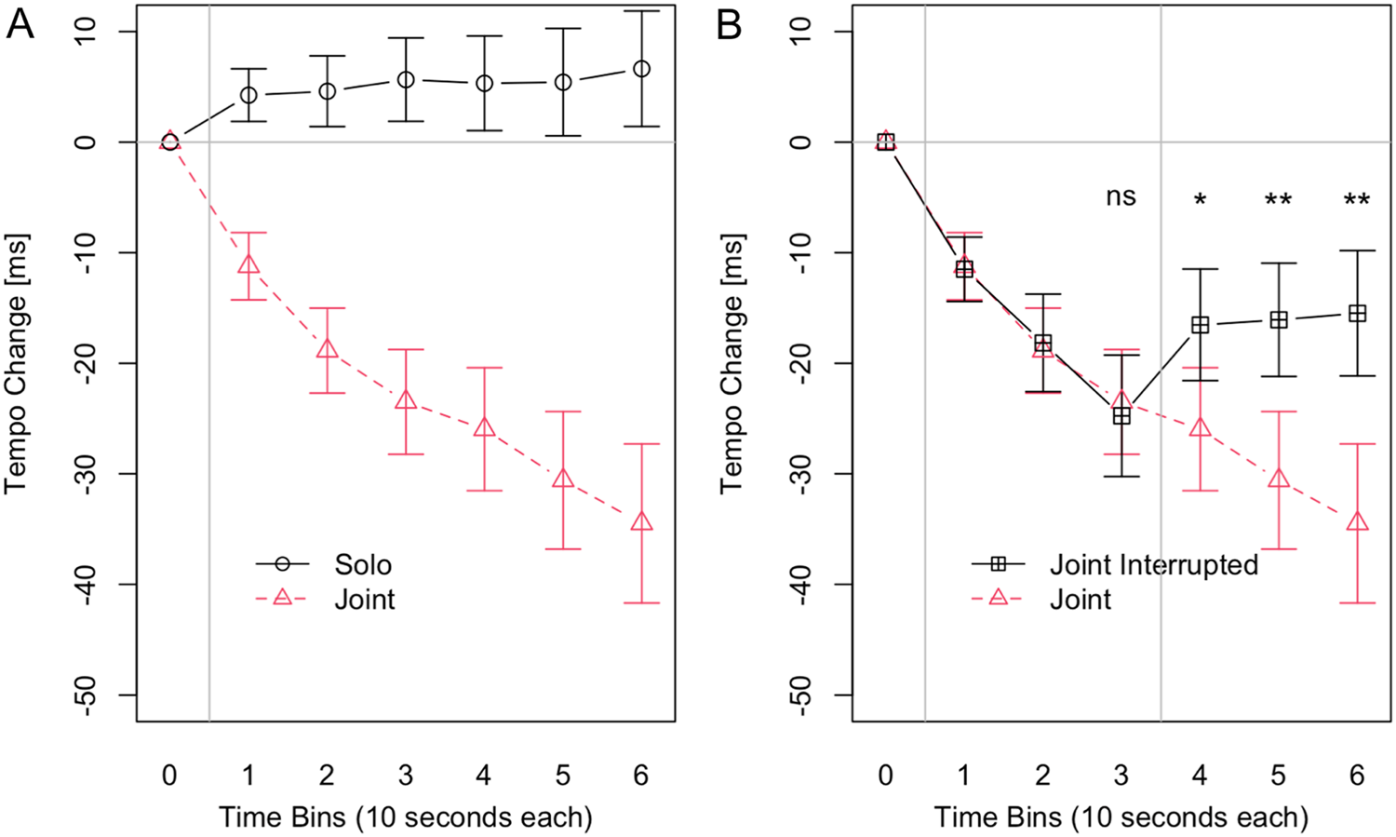
In Time Bin 0 participants synchronized with a metronome set to a target Inter-Onset-Interval of 500 ms. In Time Bins 1–6 participants were asked to maintain the initial tempo. Tempo Change on the y-axis shows how participants changed their tempo in each Time Bin compared to the initial tempo in bin 0. (**A**) Participants gradually became faster in the Joint condition but not in the Solo condition. (**B**) In the joint interrupted condition where the auditory feedback of the partner’s taps stopped after Time Bin 3 the tempo increase immediately stopped. The tempo increase continued in the joint condition in which participants continued to receive auditory feedback of the partner’s taps.

All analyses reported in this paper were computed with the R ‘ez’ package^34,35^. We entered Tempo Change in a 2 × 6 ANOVA with the within factors Task (Solo or Joint) and Time Bin (1 to 6), see Fig. 1A. The main effect for Task was significant (*F*(1, 23) = 22.097, *p* < 0.001, *η*2 = 0.297), with stronger negative Tempo Change in the Joint (mean: −24 ms, SD: 27 ms) than in the Solo condition (mean: 5 ms, SD: 20 ms). There was also a significant main effect for Time Bin (*F*(5, 115) = 8.109, *p* = 0.007, *η*2 = 0.023). The tempo increased across consecutive Time Bins (means/SDs for Time Bin 1 to 6 respectively: −3 ms/15 ms, −7 ms/21 ms, −9 ms/25 ms, −10 ms/29 ms, −13 ms/33 ms, −14 ms/37 ms). The interaction between Task and Time Bin was also significant (*F*(5, 115) = 19.990, *p* < 0.001, *η*2 = 0.033). Figure 1A shows that the tempo increased only in the Joint condition and not in the Solo condition.

To assess the effects of stopping the auditory feedback of the partner’s taps, we computed a 2 × 6 ANOVA with the within factors Feedback (Joint or Joint Interrupted) and Time Bin (1 to 6). Figure 1B shows that the tempo increase in the Joint Condition differs from the tempo increase in the Joint Interrupted condition, after the auditory feedback of the other’s taps is stopped. Accordingly, there was a significant main effect of Feedback (*F*(1, 23) = 7.54, *p* = 0.011, *η*2 = 0.020), with the overall Tempo Change being stronger in the Joint condition (mean: −24 ms, SD: 27 ms) than in the Joint Interrupted condition (mean: −17 ms, SD: 24 ms). We also found a significant main effect of Time Bin (*F*(5, 115) = 8.20, *p* = 0.003, *η*2 = 0.035), the tempo increased across consecutive Time Bins (means/SDs for Time Bin 1 to 6 respectively: −11 ms/14 ms, −19 ms/20 ms, −24 ms/25 ms, −21 ms/26 ms, −23 ms/29 ms, −25 ms/33 ms). Importantly, the interaction between Feedback and Time Bin was also significant (*F*(5, 115) = 7.54, *p* = 0.009, *η*2 = 0.025). Post-hoc comparisons, with a Bonferroni corrected significance threshold of 0.05 / 4 = 0.013, revealed significant differences in tempo increase in Time Bin 4 (*t*(23) = -2.78, *p* = 0.011, *d* = 0.568), Time Bin 5 (*t*(23) = -3.00, *p* = 0.006, *d* = 0.613) and Time Bin 6 (*t*(23) = -3.591, *p* = 0.002, *d* = 0.733) but not in Time Bin 3 (*t*(23) = 0.45, *p* = 0.660).

## Methods—Experiment 2

### Participants

We invited 24 musicians (13 women, 11 men, mean age = 29 years, SD = 10 years, mean years of musical training = 9 years, SD = 3 years and mean years of experience in music ensembles = 5 years, SD = 9 years) to participate in Experiment 2. As in Experiment 1, the sample size was determined by the same power analysis. Participants gave their informed consent and received a monetary compensation. For more information on the musician sample, please see Table 1.

**Table 1:** Detailed information about participants in Experiment 2. The questions asked in a post-experiment questionnaire were the following. What instruments do you play? How many years have you had private lessons with these instruments? How many hours per week do you currently play these instruments? How many years have you had experience teaching these instruments? If you have experience in ensemble playing, please specify which kind of ensemble and for how many years? In some cases, years of private lessons and hours of weekly practice were not provided separately for each instrument.

	Instruments	Taking lessons (in years)	Weekly playing (in hours)	Teaching (in years)	Ensemble experience (in years)
1	Violin	13	2.5	0	Orchestra (6), band (10), smaller string ensemble (occasionally)
	Piano	2	2	0	
	Guitar	1	0	0	
2	Acoustic guitar	12.5	0	0	–
3	Drums	10	8	2	Orchestra (43)
4	Guitar	15	5	25	Band, guitar ensemble (5)
5	Clarinet	6	0	0	Orchestra (5)
	Saxophone	6			
6	Piano	12	1	0	Big band (10), band (10), choir (8)
	Recorder	1	0	0	
7	Guitar, electric guitar	4	10	3	Band (12)
8	Violin, piano	7	2	0	NA (0.25)
9	Flute	7	0	0	Orchestra (3), marching band (5)
10	Saxophone	13	14	2	Band (9), orchestra (8), quartet (3)
	Piano	8	0	0	
	Guitar	3	0	0	
11	Guitar	8	4	2	Big band (1), band (6)
12	Piano, guitar	5	3.5	1	–
13	Saxophone	10	25	3	Orchestra (10), chamber ensemble (5)
	Piano	5	2	0	
	clarinet	4	5	0	
14	Piano	8	0	1	Orchestra (3)
	Flute	2	0	0	
15	Piano	6	0	1	Duos/trios (4)
	Accordion	7	0	0	
16	Piano	7	0	0	Orchestra (1)
17	Piano	5.5	1	0	–
18	Piano	8	0	0.5	Band (NA)
	Flute	1	0		
19	Piano	7	0	0	–
20	Piano	6	0	0	–
21	Flute, piano	11	1	0	–
22	Piano	13	2	0	Orchestra (2)
	Guitar	5	2		
	Erhu	2	0		
23	Flute, tuba, saxophone	6	0	0	–
24	Flute, ukulele, piano	7	1	0	–

## Results—Experiment 2

**Figure 2:**
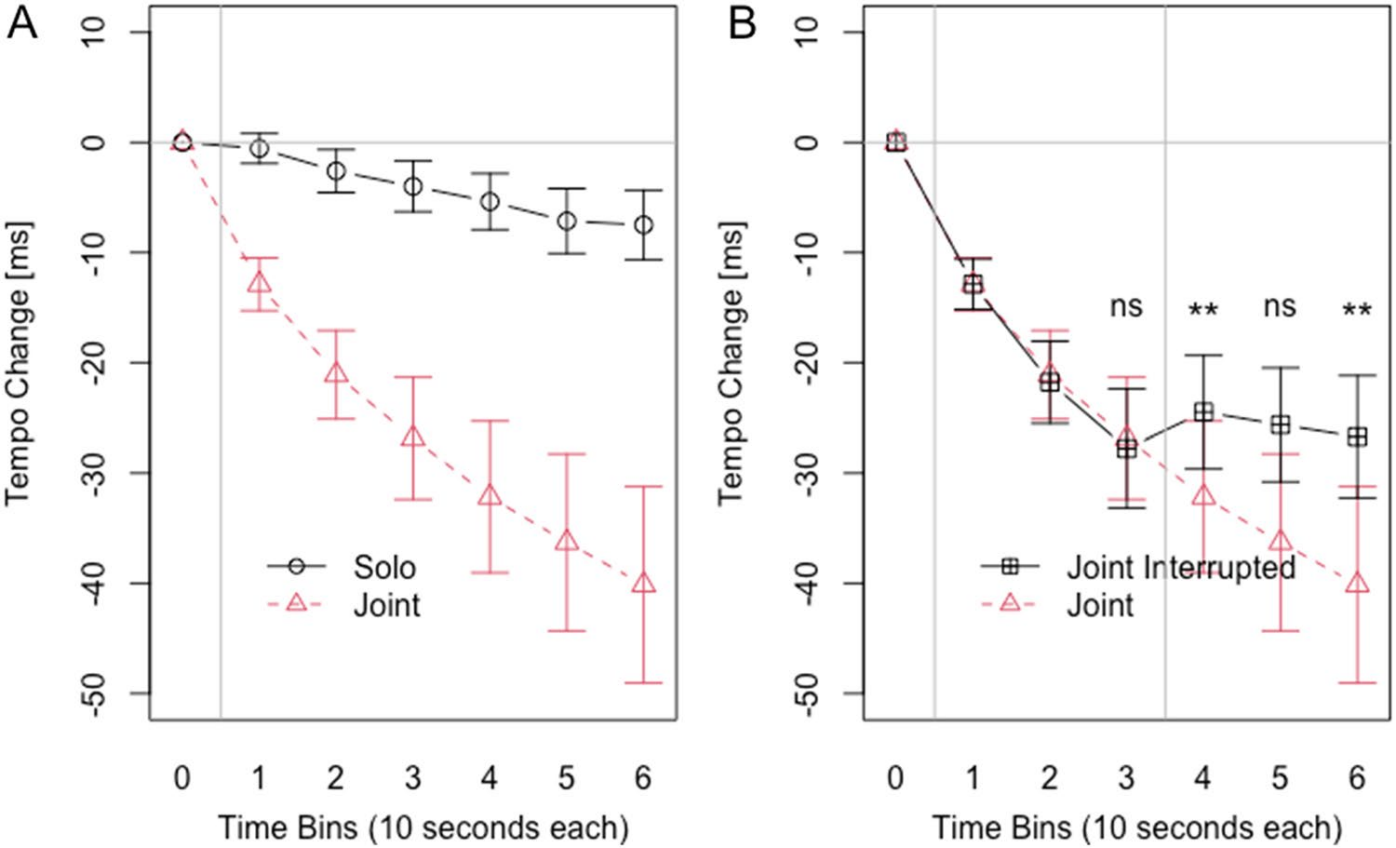
Similar to Fig. 1, but the data comes from musicians. (**A**) Participants became faster in the Joint condition than in the Solo condition. (**B**) In the joint interrupted condition where the auditory feedback of the partner’s taps stopped after Time Bin 3 the tempo increase immediately stopped. The tempo increase continued in the joint condition in which participants continued to receive auditory feedback of the partner’s taps.

We computed the same ANOVAs for musicians as we computed for non-musicians in Experiment 1, which yielded the same patterns of significance. We entered Tempo Change in a 2 × 6 ANOVA with the within factors Task (Solo or Joint) and Time Bin (1 to 6), see Fig. 2A. The main effect of Task was significant (*F*(1, 23) = 24.40, *p* < 0.001, *η*2 = 0.208), with stronger negative Tempo Change in the Joint condition (mean: −28 ms, SD: 32 ms) than in the Solo condition (mean: −5 ms, SD: 12 ms). There was also a significant main effect of Time Bin (*F*(5, 115) = 15.30, *p* < 0.001, *η*2 = 0.060). The tempo increased across consecutive Time Bins (means/SDs for Time Bin 1 to 6 respectively: −7 ms/11 ms, −12 ms/18 ms, −15 ms/24 ms, −19 ms/29 ms, −22 ms/33 ms, −24 ms/36 ms). The interaction between Task and Time Bin was also significant (*F*(5, 115) = 12.39, *p* = 0.001, *η*2 = 0.021). Figure 2A shows that the tempo increased in the Joint condition differs from the tempo increase in the Solo condition.

To assess the effects of the stopping the auditory feedback of the partner’s taps, we computed a 2 × 6 ANOVA with the within factors Feedback (Joint or Joint Interrupted) and Time Bin (1 to 6). Figure 2B shows that the tempo increase in the Joint condition differs from the tempo increase in the Joint Interrupted condition, after the auditory feedback of the other is stopped. Accordingly and as with saw in the data from non-musicians, there was a significant main effect for Feedback (*F*(1, 23) = 5.84, *p* = 0.024, *η*2 = 0.009) with the overall Tempo Change being stronger in the Joint condition (mean: −28 ms, SD: 32 ms) than in the Joint Interrupted condition (mean: −23 ms, SD: 23 ms). There was also a significant main effect of Time Bin (*F*(5, 115) = 14.93, *p* < 0.001, *η*2 = 0.061) as the tempo increased across consecutive Time Bins (means/SDs for Time Bin 1 to 6 respectively: −13 ms/11 ms, −21 ms/19 ms, −27 ms/26 ms, −28 ms/30 ms, −31 ms/33 ms, −33 ms/37 ms). Importantly, there was also a significant interaction (*F*(5, 115) = 9.40, *p* = 0.003, *η*2 = 0.012). Post-hoc comparisons, with a Bonferroni corrected significance threshold of 0.05 / 4 = 0.013, revealed a significant difference in Time Bin 4 (*t*(23) = -2.88, *p* = 0.008, *d* = 0.588) and most importantly in Time Bin 6 (*t*(23) = -2.95, *p* = 0.007, *d* = 0.602), but not in Time Bin 3 (*t*(23) = 0.73, *p* = 0.473) and Time Bin 5 (*t*(23) = -2.59, *p* = 0.016, *d* = 0.530).

### Comparing musicians to non-musicians

**Figure 3:**
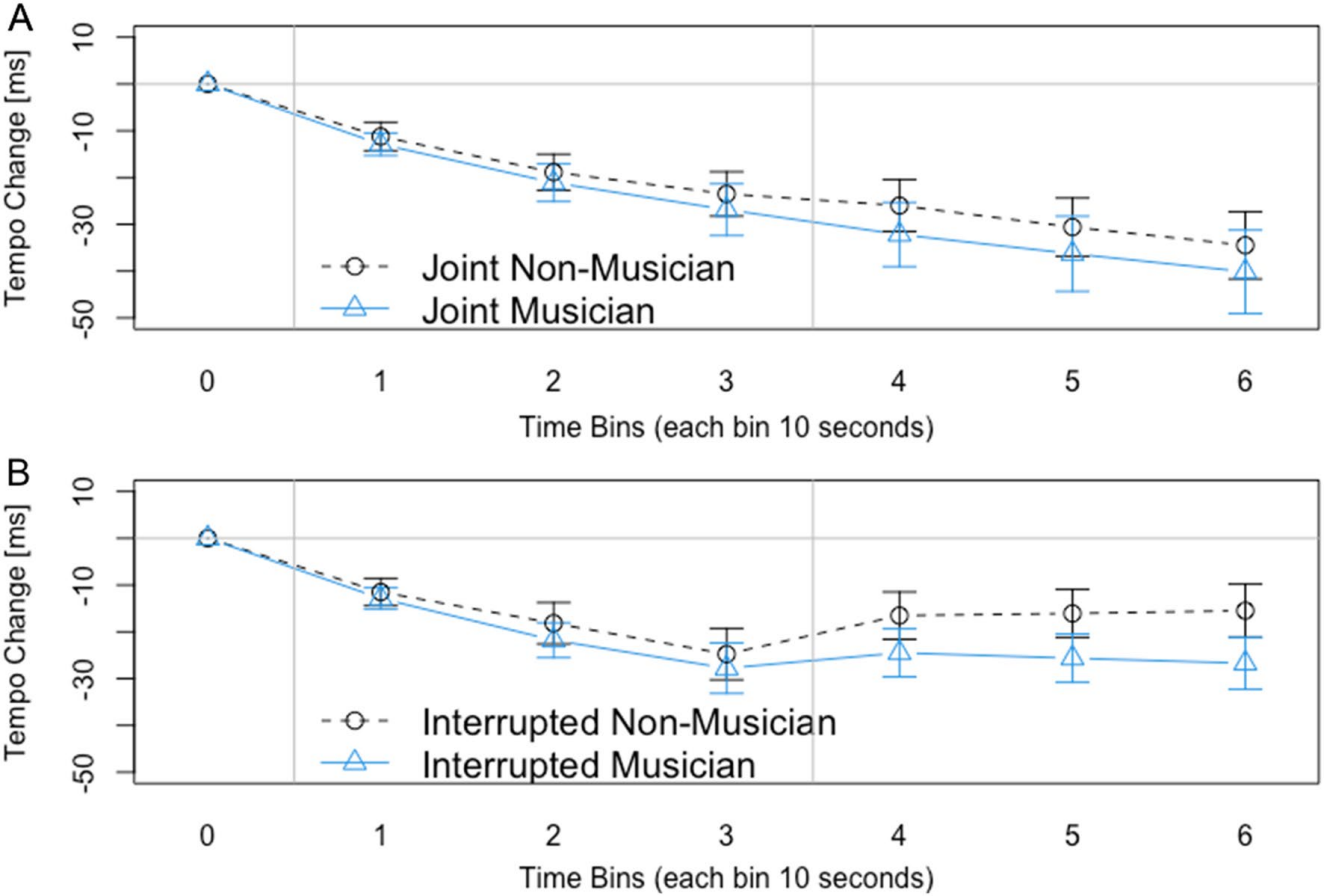
A direct comparison between Musician and Non-Musician data. (**A**) The Tempo Change in the Joint condition which did not differ between Non-Musicians and Musicians. (**B**) The Tempo Change in the Joint Interrupted condition for Non-musicians and Musicians. There was also no significant difference between the two groups in this condition.

We directly compared the effects of Joint Rushing between musicians and non-musicians. To investigate whether the pattern of Joint Rushing differed between musicians and non-musicians, we computed a 2 × 6 ANOVA with the factors Musician (Musician or Non-Musician) and Time Bin (1–6) for the Relative ITIs in Joint trials. There was a significant main effect of Time Bin, *F*(5, 230) = 32.38, *p* < 0.001, *η*2 = 0.082, but neither a main effect of Musician, *F*(1, 46) = 0.29, *p* = 0.596, nor a significant interaction, *F*(5, 230) = 0.37, *p* = 0.559. Thus, there was no indication that musicians showed less of a tempo increase than non-musicians (see Fig. 3A).

To investigate whether musicians were better able to recover from effects of joint rushing after the feedback from the partner was stopped, we computed a 2 × 4 ANOVA with the factors Musician (Musician or Non-Musician) and Time Bin (3–6) for the ITIs from Joint Interrupted trials. There was a significant main effect of Time Bin, *F*(3, 138) = 3.84, *p* = 0.049, *η*2 = 0.009, but neither a main effect of Musician, *F*(1, 46) = 1.24, *p* = 0.271, nor a significant interaction, *F*(3, 138) = 1.61, *p* = 0.189. There was no indication that in the joint interrupted condition musicians were more likely than non-musicians to return to the initially instructed tempo (see Fig. 3B).

**Figure 4:**
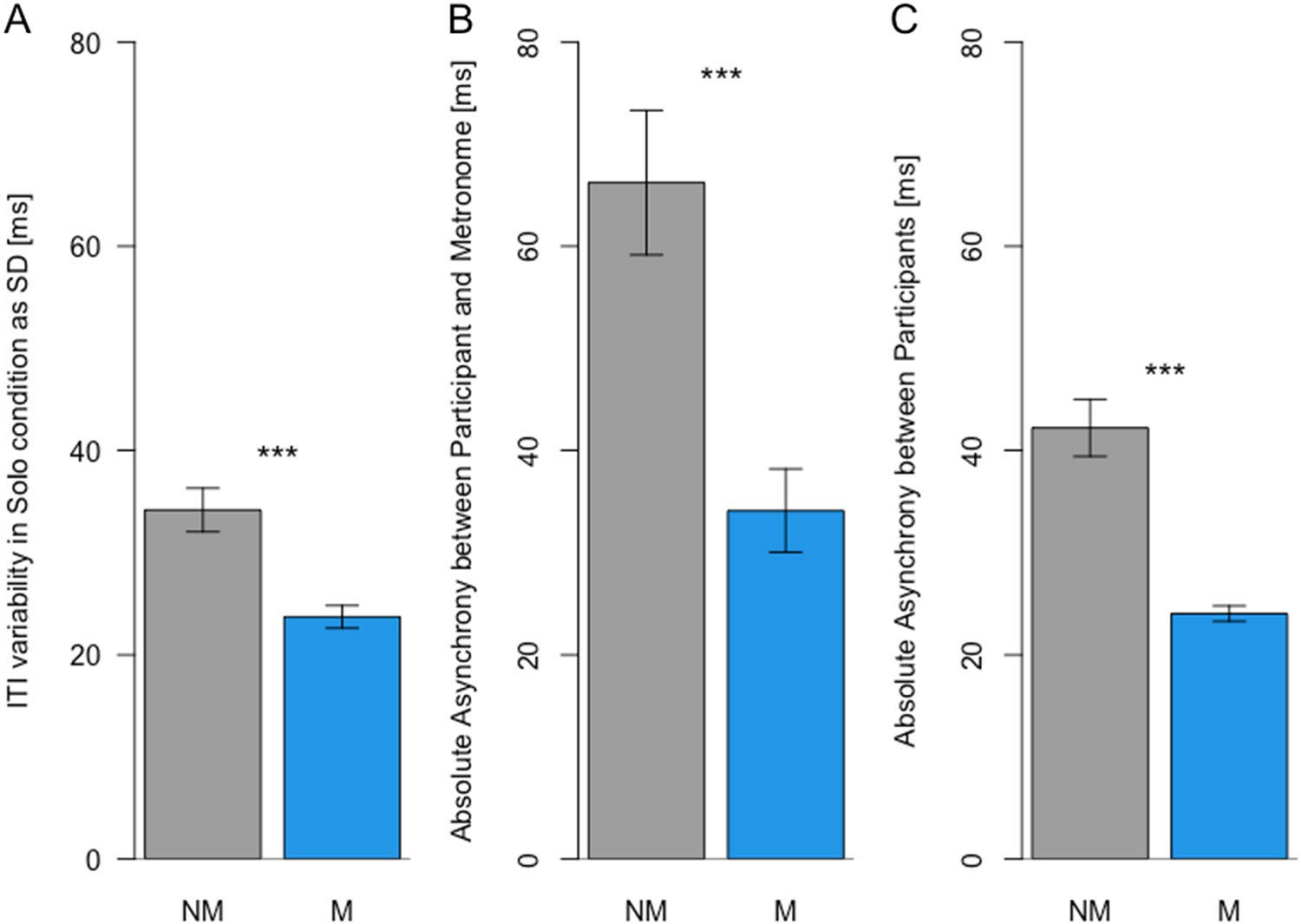
The difference between Non-Musicians (NM) and Musicians (M) for three different measures of timing performance. (**A**) The difference in ITI variability, which was computed from all trials in the Solo condition. (**B**) The difference in absolute asynchrony which was calculated on data from the Time Bin 0 of all trials, as Time Bin 0 was the same, i.e., participants heard and synchronized to a metronome, in all conditions. (**C**) Absolute asynchrony as computed on all trials from the Joint condition, in which participants could hear each other.

We also assessed whether musicians differed from non-musicians with respect to their sensorimotor synchronization abilities. To do so, we computed the variability of ITIs in the Solo condition as standard deviations, which provides us with an indication of how stable a participant’s tapping performance was, the absolute mean asynchronies with the metronome during the synchronization phase (first 10 s of each trial), which provides us with an indication of how good participants are at sensorimotor synchronization with a stable timekeeper, i.e., a metronome, and we estimated the asynchrony between participants in trials from the Joint condition, which indicates how well participants were able to synchronize with an adaptive partner. Three Bonferroni corrected t-tests (significance level p = 0.017) revealed that musicians differed significantly from non-musicians on all three measures. Musicians showed significantly lower ITI variability in Solo trials (mean = 24 ms, SD = 5 ms) than non-musicians (mean = 34 ms, SD = 10 ms), *t*(34.58) = 4.35, *p* < 0.001, *d* = 1.254 (see Fig. 4A). Musicians showed significantly lower absolute asynchronies with the metronome (mean = 34 ms, SD = 20 ms) than non-musicians (mean = 66, SD = 35), *t*(36.64) = 3.93, *p* < 0.001, *d* = 1.136 (see Fig. 4B). Musicians also showed significantly lower absolute interpersonal asynchronies (mean = 24 ms, SD = 4 ms) than non-musicians (mean = 42 ms, SD = 14 ms), *t*(26.43) = 6.26, *p* < 0.001, *d* = 1.808 (see Fig. 4C).

### A closer look at musicians’ performance

**Figure 5:**
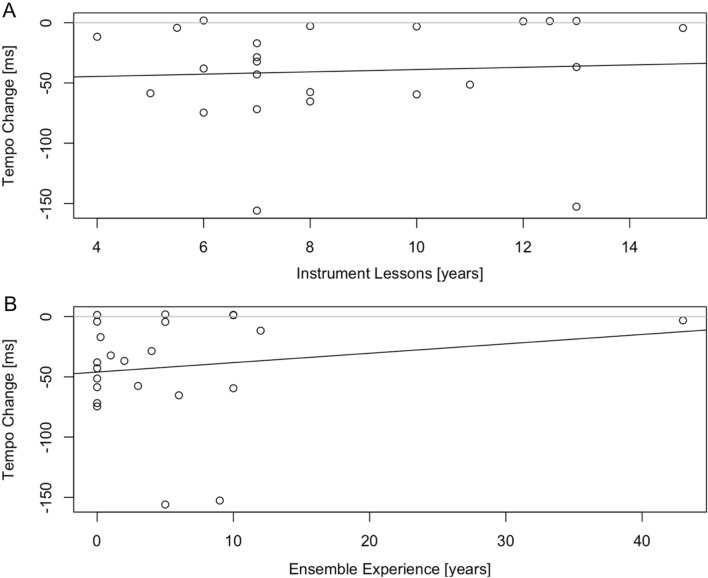
Each musicians’ tempo change as a function of years of private lessons on their main instrument (**A**) and years of experience with ensemble playing (**B**). The black lines represent the results of the corresponding linear regressions. Neither regression was significant.

Finally, we assessed whether the years of training musicians had on their instrument or the years of experience with ensemble playing musicians had correlates with their joint rushing performance. To do so, we computed a linear regression in which we used *years of private lessons on main instrument* as a predictor for joint rushing. Results show that years of private lessons did not predict tempo change, *R*^2^ = 0.00, *F*(1, 21) = 0.10, *p* = 0.756 (see Fig. 5A). We also computed a linear regression in which we used *years of experience with ensemble playing* as a predictor for joint rushing. Results show that years of ensemble experience did not predict tempo change either, *R*^2^ = 0.03, *F*(1, 21) = 0.57, *p* = 0.459 (see Fig. 5B).

## Discussion

The aim of the present study was to test whether joint rushing is caused by the same error correction mechanisms that support temporal coordination. The first prediction we tested was that joint rushing should depend on continuous input from a partner’s signal. The results of two experiments clearly support this prediction because they demonstrate that rushing does not continue when perceptual input from the partner becomes unavailable. This is in line with joint rushing models that are based on error correction mechanisms that perform adjustments based on the timing of a person’s own action and the timing of external signals^10,16,17^. The results did not provide any evidence that joint rushing is caused by arousal or anxiety^17,25^ or using internal models to keep simulating the partner’s action timing, once the feedback is interrupted^29–31^. Future studies could incorporate physiological measurements such as heart rate and skin conductance to rule out the possibility that arousal or anxiety levels fluctuated in synchrony with the auditory feedback manipulation.

The second prediction we addressed in the present study was that joint rushing affects the period of an internal timekeeper. The results of two experiments also support this second prediction. After perceptual input from a partner had stopped, participants continued to tap at an increased speed. There was no indication of a rebound to the initial tempo which would be expected if the period of the internal timekeeper remained unaffected by joint rushing. This finding is important, because models based on the canonical period correction mechanism^26^ would predict a return to the initial tempo.

Based on previous tapping experiments, in which participants had to synchronize with tempo changing metronomes, canonical period correction was described as an intentional, attention-requiring mechanism that corrects an internal timekeeper in a stepwise fashion^26^. During joint rushing however, tempo changes are unintended, below perceptible levels and continuous. Thus, the present results indicate that the period of an internal timekeeper can be adjusted in an unintentional manner that does not seem to require attention. An interesting question for future research is whether and how trying to achieve temporal coordination with another person increases the likelihood for automatic period adjustments of an internal timekeeper.

Our findings imply that the flattening of the tempo curve during joint rushing cannot be due to a lack of period correction. This flattening refers to the finding that joint rushing is more pronounced at the beginning of a joint rhythmic performance and less pronounced or absent during later parts of the performance. One possible explanation for this flattening is that tempo changes go unnoticed early on in a joint performance but become noticeable later on. Once an increase in tempo is noticed period correction kicks in and prevents further tempos increase. In line with such an explanation Okano et al.’s model^17^ includes a term that represents an intention to maintain an initial tempo. This term scales with the difference between the initial tempo and the current tempo, such that the larger the difference between the two the larger the intention to maintain the initial tempo. However, our findings provide clear evidence that the initial tempo is not maintained internally. Rather, unintentional period correction seems to occur even at early stages of a joint performance (30 s in the present experiment). Hence, it remains an open question why tempo increases flatten out, as joint rhythmic performance progresses. It also remains an open question whether joint rushing is functional in some sense. For example, if joint rushing serves the function of bringing rhythmic interactions up to a tempo that facilitates smooth coordination^10^, the flattening out at a certain tempo is an important feature, as otherwise the interaction would accelerate until individual performance limits are reached.

The third question we addressed in the present study is whether expertise with timing and temporal coordination reduces rushing during joint rhythmic performances. The results for musicians were the same as for non-musicians. Musicians’ tempo increase in the joint condition was comparable to non-musicians’. The results of two linear regression analyses further showed that the joint rushing performance in musicians was neither a function of years of musical training nor of years of experience with ensemble playing. Thus, musical expertise did not help with reducing or preventing joint rushing. This finding provides a further indication that joint rushing is caused by an automatic mechanism that cannot be controlled consciously.

There was also no indication that musicians were more likely to return to their initial tempo. Hence, musicians could not resist the adjustments of the period of a timekeeper that occurs during joint rushing. This result seems to be incompatible with Thomson et al.’s^9^ finding of no rushing in musicians. However, it is important to remember that these findings were obtained with a group of students of a rhythm class. Thus, it could be that musicians are able to avoid joint rushing in large groups but not in small groups. Alternatively, rhythm combos may use specific timing strategies to avoid joint rushing.

The social interaction in this study was purely based on the exchange of auditory signals. Like most studies on temporal coordination, we focused on audition because the temporal resolution of audition in humans is higher than the temporal resolution of other senses, such as vision or touch^36^. Thus, whenever a joint action also produces audible effects (dancing, walking together etc.) phenomena such as joint rushing will probably be driven by audition. Accordingly, in a previous study, in which participants could hear and see each other in the joint condition we found similar levels of joint rushing as in the present study^10^. For joint actions where auditory information is absent or very noisy, we would expect reduced accuracy and higher variability of temporal coordination due to the lower temporal resolution of the visual system. Based on the findings that joint rushing is positively correlated with variability^9^, this would lead to the prediction that there could be even more rushing when temporal coordination must be achieved through the visual modality.

In more general terms, our results add to previous studies demonstrating that tempo changes in social interactions are not a “methodological nuisance” but allow us to better understand mechanisms of temporal coordination. An important question for future research is whether such systematic tempo change during social interaction is more than a problem with keeping in time together. Such tempo changes could play an important role in facilitating coordination. For instance, joint rushing could function as a coordination smoother^37^ that brings the tempo of an interaction to an optimal balance between individual performance limits and the needs of temporal coordination. One way to address this question, would be to further study how individual performance limits and the needs of temporal coordination affect the flattening of the tempo curve. Are there situations where joint rushing continues until the performance cannot be maintained any longer (think about dances like Sirtaki)? How is joint rushing related to the physical performance constraints of the individual actors? Is joint rushing pulling towards an optimal tempo for the given interaction? Is there automatic joint slowing if this is beneficial for an interaction? Addressing these questions will enhance our understanding of rhythmic joint actions, from making music together to coordinated manual labor.
